# When Words Shape Care: Educating First-Year Medical Students on Identifying and Replacing Bias in Electronic Health Documentation

**DOI:** 10.15766/mep_2374-8265.11646

**Published:** 2026-10-29

**Authors:** Becka Konnayil, Sravya Varanasi, Christina Jacob, Pinhong Wu, Cordia Beverley, Andrew Wackett

**Affiliations:** 1 Medical Student, Renaissance School of Medicine at Stony Brook University; 2 Medical Student, Renaissance School of Medicine at Stony Brook University; 3 Medical Student, Renaissance School of Medicine at Stony Brook University; 4 Medical Student, Renaissance School of Medicine at Stony Brook University; 5 Clinical Associate Professor, Department of Family, Population, and Preventive Medicine, Renaissance School of Medicine at Stony Brook University; 6 Clinical Associate Professor, Department of Emergency Medicine, Stony Brook University Hospital

**Keywords:** Bias Recognition, Case-Based Learning, Charting Skills, Clinical Documentation, Health Disparities, Health Equity, Patient Records, Stigmatizing Language

## Abstract

**Introduction:**

Although medical education increasingly addresses bias in patient care, less attention has been paid to biased language in clinical documentation within the Electronic Health Record (EHR), a critical concern as patients have increasing access to their records. Because biased language can influence clinicians’ perceptions and negatively affect patient relationships, focused medical student training is needed to promote equitable, patient-centered care.

**Methods:**

We implemented a one-hour workshop on biased language in medical records using case-based discussions. First-year students (MS1s) completed pre- and postsurveys assessing their ability to identify biased language. Results were analyzed using paired *t*-tests, descriptive statistics, and thematic analysis.

**Results:**

Sixty-nine MS1s attended the workshop; 68 completed the presurvey (98.5%), and 63 the postsurvey (91.3%). Students demonstrated significant improvement in their ability to identify biased language in patient cases (*P* < .001) and survey scores (*P* < .001). Significant improvements were also observed in students’ ability to identify the word “claims” as biased language (*P* < .001), identify language that defined patients by their illness (*P* < .001), and recognize negative patient descriptions (*P* < .001). Students valued the workshop's use of examples of biased language and neutral language alternatives, and many recommended incorporating this training into the medical school curriculum.

**Discussion:**

The workshop improved MS1s’ recognition of biased language in EHR documentation. Incorporating documentation bias training early in medical school may strengthen students’ awareness of biased language and support more patient-centered documentation.

## Educational Objectives

By the end of this activity, learners will be able to:

1. Describe the potential impact of stigmatizing and biased language on provider perceptions and patient care.
2. Identify biased language commonly used in medical documentation.
3. Employ patient-centered, neutral, and acceptable alternatives to biased or stigmatizing language in clinical notes.

## Introduction

Health care disparities persist across the United States, contributing to adverse patient outcomes and experiences. One mechanism by which these disparities are perpetuated is biased language in medical documentation in the Electronic Health Record (EHR), which may characterize patients based on race, medical condition, disability, or other social and clinical attributes.^1-3^

Biased language in medical records influences both provider perceptions and clinical decision making. Goddu et al. demonstrated that medical students and residents exposed to documentation describing a patient with sickle cell disease as a “sickler” were less likely to recommend opioid analgesia and more likely to report negative impressions of the patient.^4^ Another study found that mental health professionals were more likely to make character-based judgments and recommend punitive measures when patients were described as “substance abusers” rather than as individuals with substance use disorders.^5^

With expanded access to EHRs, patients and their families are more likely to read their clinical notes. Patients exposed to stigmatizing language in their charts report lower ratings of provider attitudes and diminished trust in their clinicians. Pont et al. described that such language worsens feelings of shame, and others have shown that biased language reduces patient engagement in both treatment and preventive care.^4,6-8^ Common documentation phrases such as “patient claims” may make patients feel the validity of their experiences is being questioned.^9^ Biased verbal handoffs are also associated with decreased accuracy of clinical information recall.^10^ Patients have also turned to social media to highlight stigmatizing language in their records.^11^

Undergraduate medical education (UGME) environments often normalize biased language through the “hidden curriculum,” the discrepancy between formal instruction and the informal lessons absorbed through clinical culture and role modeling.^12-14^ Although medical school curricula increasingly emphasize diversity, equity, and bias recognition, learners may continue to adopt stigmatizing documentation practices. As a result, educators have called for earlier and formal instruction focused on humanism, ethics, and patient-centered communication.^15^

Several educational workshops have been developed to address biased language. Stagno et al. implemented a documentation-focused workshop in a mental health context to promote patient-centered communication.^16^ Mayfield et al. developed training for medical students to improve inclusive language during sexual history taking with LGBTQ+ patients.^17^ Raney et al. created an interactive, interdisciplinary workshop aimed at addressing stigmatizing language.^18^ While these initiatives demonstrate the importance of teaching bias-aware communication, few have specifically evaluated learners’ ability to identify stigmatizing language within medical documentation through direct pre- and postintervention assessment.

To address these gaps, we developed a case-based charting workshop for first-year medical students (MS1s) focused on identifying biased language in medical records and recognizing patient-centered alternatives. Rather than a multicomponent antibias training, the workshop was intentionally designed as a brief one-hour session requiring no participant preparation, intended to supplement existing preclinical note-writing instruction. In contrast to Raney et al.’s workshop, which was developed for practicing health care professionals whose documentation habits were already shaped by years of clinical experience, our workshop was specifically designed for MS1s so this content could help students develop reflective documentation habits before clinical training, where existing norms may be reinforced.^18^ By reaching learners at the moment they are composing their first clinical notes, the workshop aims to shape documentation habits as they form, rather than asking learners to unlearn language. Workshop materials emphasized the impact of biased documentation on patient care and provider perceptions, with students reviewing case examples in both small- and large-group formats to encourage active discussion. To address Raney et al.’s limitation of not concretely assessing knowledge improvement, we utilized pre- and postworkshop surveys to directly measure students’ ability to recognize stigmatizing language in clinical documentation.^18^

## Methods

### Activity Design and Development

After reviewing existing preclinical and clinical curricular content at the Renaissance School of Medicine (RSOM), we identified a lack of explicit guidance on bias-aware documentation. MS1s begin writing clinical notes during the course Introduction to Clinical Medicine (ICM), with instruction focused on content detail and proper note structure rather than language choice. Students are educated about the effects of biased language on patient care, but are not instructed on which language to avoid, leaving them without concrete examples of changes they can make to their documentation skills. We chose MS1s as our demographic of interest because their minimal experience in note writing meant we could focus on preventing the development of biased language habits, rather than unlearning previous training.

We developed the workshop using Kern's six-step approach to curriculum development, with the identified gap serving as our general needs assessment.^19^ The development team consisted of two medical students and teaching faculty at RSOM. We began by defining biased language in documentation as words or phrases that (1) signal doubt about a patient's experience (eg, “claims,” “believes”); (2) convey stereotypes on the basis of race/ethnicity, chronic conditions, age, mental health, or substance use (eg, “schizophrenic,” “substance abuser,” “wheelchair bound”); (3) attribute negative characteristics to a patient's behavior (eg, “noncompliant,” “frequent flyer”); or (4) introduce medically irrelevant details that may prejudice readers (eg, incarceration history).^3-5,9^

Raney et al.’s antibias workshop was a useful resource for developing our workshop. Specifically, we appreciated their use of clinical vignettes, as at RSOM, students learn note writing by reading examples of patient cases. We also adopted small- and large-group discussions from Raney et al.’s workshop, as their participants’ feedback indicated appreciation of the small-group format.^18^

The case examples used in our workshop slides and surveys were generated by referencing EHR note examples from RSOM's ICM course materials, with language altered to include examples from each category of our definition of biased language.^4,5,9,11,16,18^ Each case included multiple categories of biased language and was designed to be easily understandable without significant medical knowledge. Emphasis was given to language that the development team had encountered in clinical practice, such as “sickler” and “claims,” to expose students to language they were most likely to encounter. The session was organized to last one hour to ensure adequate time for group discussion, without overwhelming students. Finalized material went through faculty review by professors in RSOM's Department of UGME and the Department of Family, Population, and Preventive Medicine.

### Curricular Context and Facilitator Preparation

ICM is a required longitudinal first- and second-year course, in which all students (138 in the Class of 2028) learn history taking, physical examination, and clinical note writing. Our audience of interest was MS1s just beginning to perform history and physical examinations. We implemented the workshop twice as an additional activity after students’ required ICM sessions. The workshop required no prerequisite knowledge, preparation, or prior coursework on documentation language, making it accessible to learners from all backgrounds.

Two second-year medical students who helped develop the workshop materials facilitated the sessions without a faculty member present. Both facilitators reviewed the appendix materials (slide deck, patient cases, facilitator's guide, and surveys) and completed a practice session with faculty before delivery, requiring approximately 2 hours of preparation. Facilitators were encouraged to draw on their own clinical experiences with documentation language to prompt discussion.

### Implementation

Participation in the charting workshop was voluntary as the activity was not a required component of the curriculum; we offered no incentives, and nonparticipation did not affect students’ grades. Workshop information was distributed via email to the first-year class, which included the presurvey to be completed before attending the session.

The workshop was held in a lecture room designed to allow students to easily transition between large- and small-group discussions. Two facilitators first presented an overview of biased language and its potential impact on patient care and provider perceptions, followed by examples of commonly used biased phrases and patient-centered alternatives (Appendix A). In small groups, participants were asked to identify examples of biased language in the displayed patient case vignettes (Appendix B) and propose neutral language alternatives. After each case, students volunteered to share their thoughts with the entire group. Before ending, students received a link to the postworkshop survey, which was also emailed the following day. The presentation content, including the order of activities, corresponding slides, and presentation script, was organized into a guide for facilitators interested in conducting a similar workshop in the future (Appendix C).

### Assessment and Evaluation

As prior studies have limited their evaluations to participant self-reported awareness, we chose our questions to directly assess students’ knowledge growth and, therefore, the effectiveness of our workshop design. A member of the development team developed the survey instruments, mapped to each of the educational objectives. Each survey was reviewed for language clarity, content, and face validity by an independent statistician. Following completion of the initial statistical analysis, the approach, results, and their interpretation were reviewed by the same statistician.

Each survey had two patient case examples, in which learners were required to identify specific terms that reflected biased language, and four general-knowledge questions (true/false and “select all that apply”) assessing participants’ understanding of the effect of biased language on a patient's care and who can be exposed to biased language in a note. The presurvey gathered demographic information, including race and gender, to explore whether recognition differed across groups. The postsurvey included three 5-point Likert-scale items on session utility and attendees’ confidence in identifying biased language, as well as two open-ended feedback items.

Pre- and postsurvey cases were different scenarios so that improvement could not reflect recall of specific answers. While no formal method was used to match the surveys on difficulty, each survey was designed to include language that drew equally from each of the four defined categories of biased language, as well as language explicitly identified as biased during the workshop.

We invited all MS1s to complete the preworkshop survey (Appendix D), though they were not required to do so to attend the workshop. Immediately following the session, we instructed workshop participants to complete the postworkshop survey (Appendix E). Surveys were anonymous and administered via Qualtrics. To allow matching of pre- and postsurvey responses, participants created a unique identifier used across surveys.

### Statistical and Qualitative Analysis

The two medical students on the development team assigned scores using a scoring rubric that gave students one point for each correct identification of biased language as “inappropriate” and nonbiased language as “appropriate,” with no points gained or lost for selecting an incorrect answer or “unsure.” We calculated case (total score: presurvey = 12; postsurvey = 11), general-knowledge (total score = 7), and overall scores (total score: presurvey = 19; postsurvey = 18) for each participant. We analyzed quantitative data using paired *t*-tests to compare pre- and postsurvey scores. We also used paired *t*-tests to assess changes in the identification of specific types of bias, including the use of “claims,” defining patients by their illness, and negative descriptions of patient behavior. Likert-scale responses were summarized using means, standard deviations, and ranges.

Our primary outcome, identification of biased language, was scored against a fixed answer key, so the collected data are less susceptible to response-shift bias than a self-report measure would be. The Likert items assessing students’ confidence in identifying biased language and understanding its effects are self-rated but were not collected in the presurvey. Their analysis was not used to assess students’ change in confidence as a measure of the workshop's effectiveness, but rather if students qualitatively felt that the workshop was helpful.

We analyzed the qualitative data from open-ended survey responses using thematic analysis. The two development team members independently coded the responses using an inductive approach, then met to reconcile differences and develop a shared codebook. Intercoder reliability was strong, with a percent agreement of 92.3% and a Krippendorff's alpha of 0.914 for nominal data, calculated after excluding nonsubstantive responses. We resolved any discrepancies by consensus and organized codes into overarching themes.

We conducted all quantitative analyses using IBM SPSS Statistics (version 29.0). This project was deemed exempt by the RSOM Institutional Review Board (IRB2024-00510).

## Results

A total of 69 (50%) MS1s attended the charting workshop. The cohort who completed the presurvey (*n* = 68) included 22 (32.4%) male and 46 (67.6%) female students. Self-identified race and ethnicity included 40 (58.8%) Asian, 20 (29.4%) White, one (1.5%) Hispanic or Latino, and seven (10.3%) multiracial students (six Asian and White, and one Hispanic or Latino and White). Analysis of demographic data showed no statistically significant differences in responses by race or gender.

When comparing pre- and postsurvey (*n* = 63) results, there was a statistically significant difference in scores on the patient cases (*P* < .001) and the overall score (*P* < .001), as shown in Table 1. There was also a statistically significant improvement in scores for general knowledge of biased language (*P* = .001).

**Table 1.**
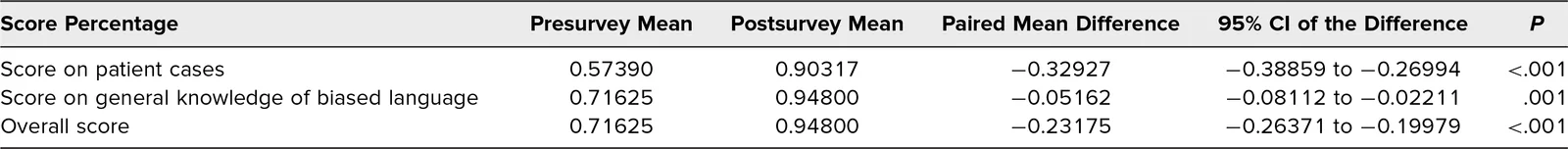
Paired *t*-Tests Comparing Pre- and Postsurvey Responses

When comparing specific scores between pre- and postsurvey responses, there was a statistically significant increase in correct identification of all the following as biased language: the use of “claims” to indicate what the patient says, defining the patient by their illness, and negative descriptions of patient behavior, as shown in Table 2.

**Table 2.**
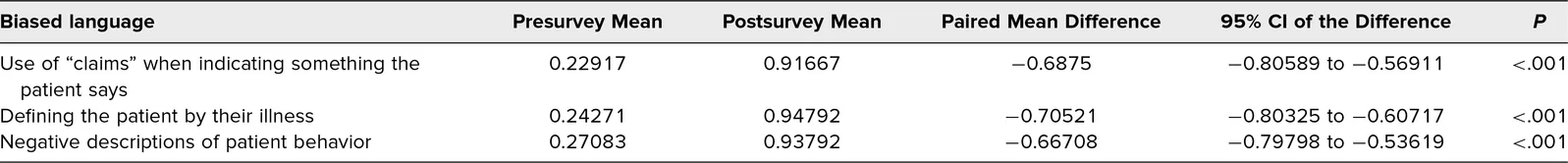
Paired *t*-Tests Comparing Pre- and Postsurvey Responses for Specific Biased Language

The postsurvey asked students to reflect on the utility of the workshop and self-rate their confidence in their abilities to identify biased language. Respondents (*n* = 46) agreed that the workshop was helpful (*M* = 4.9130; *SD* = 0.28488), gave them a better understanding of what biased and unbiased language is (*M* = 4.8696; *SD* = 0.34050), and taught them why biased language can impact a patient's care even years down the line (*M* = 4.9783; *SD* = 0.14744).

As shown in Table 3, many students reported increased understanding of what constitutes biased language, such as “claims” or “sickler.” Several responses highlighted how seemingly neutral documentation may shape clinical perceptions. Participants further emphasized that respectful terminology in EHR evolves and requires ongoing reflection. Participants raised several future directions to refine the workshop, as detailed in Table 3. Many expressed support for integrating documentation bias training into the formal curriculum, such as RSOM's ICM course. Participants also offered specific recommendations for enhancing the workshop, including providing handouts or slide decks for reference, incorporating additional real-world cases, and discussing examples in which biased documentation negatively affected patient care.

**Table 3.**
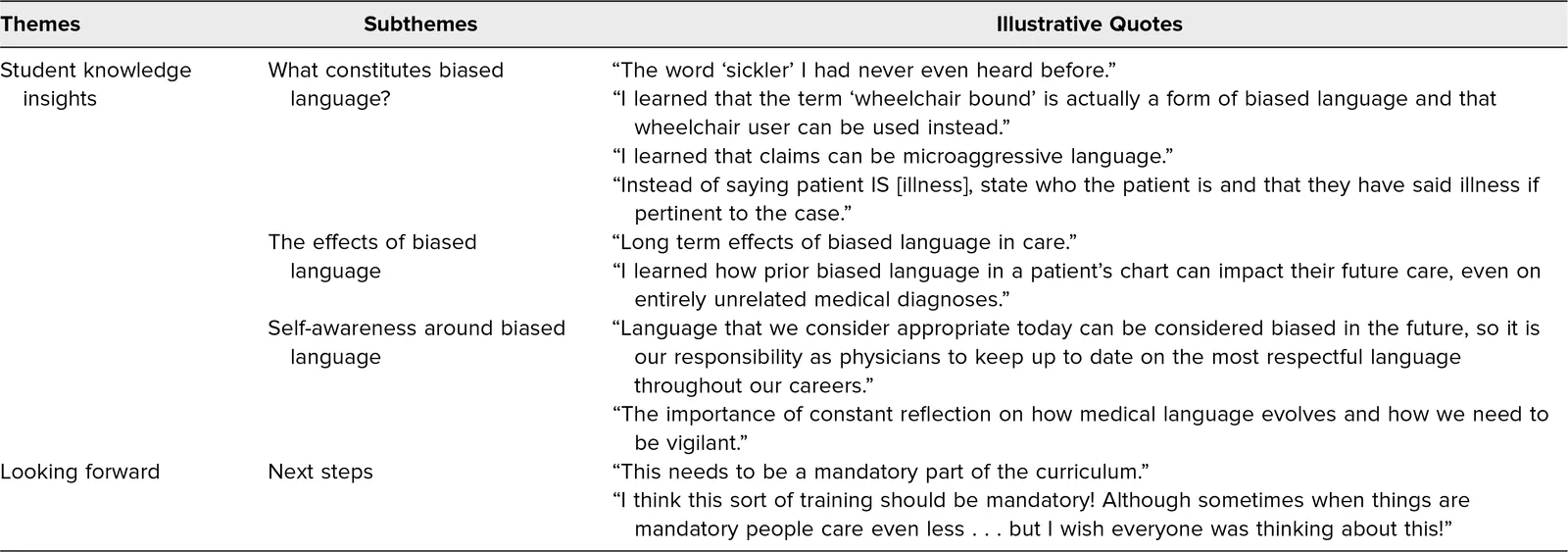
Themes From Postworkshop Survey's Open-Ended Responses (*N* = 63)

## Discussion

Participants in this workshop showed measurable gains in their ability to recognize the effects of biased language on patient care and to identify biased language in clinical notes, demonstrating support for educational objectives 2 and 3. Support for educational objective 1 was assessed qualitatively through open-ended survey responses, which revealed that many students reported increased awareness of how seemingly neutral language can perpetuate bias and influence how patients are perceived within medical teams.

A distinguishing feature of our workshop design was the assessment of skill growth via pre- and postsurveys. Existing curricula addressing stigmatizing language have largely evaluated participant self-perception of their awareness or confidence, and Raney et al.’s antibias workshop noted that the absence of a pre-session assessment prevented them from commenting on knowledge change.^18^ We addressed this gap by comparing student performance before and after the workshop, allowing us to assess whether the workshop truly increased student ability to identify biased language. Additionally, the small- and large-group formats allowed for thoughtful discussion of patient cases and constructive feedback from peers and workshop moderators.

Many students advocated for incorporating the workshop into the ICM curriculum as a required session for all MS1s. Participants also asked for real-world examples in which biased documentation affected patient care, suggesting that direct patient accounts would better demonstrate the effects of biased language than data alone. Others requested materials to reference while charting; the “dos and don'ts” slides in the slideshow presentation (Appendix A) could be converted into a handout that can be distributed at the end of the session.

### Limitations

The workshop survey instruments were reviewed for content validity but were not formally assessed for reliability or internal consistency. Additionally, although different cases were used in the pre- and postsurveys to avoid memorization, we did not formally validate them as equivalent in difficulty. Improvement may partially reflect the testing effect or priming from the presurvey, rather than the workshop itself. However, because the primary outcome of correct identification of biased language was scored via a fixed answer key, it is less vulnerable to response-shift bias. Participation in the workshop and pre- and postsurveys was voluntary, introducing potential selection bias, and baseline familiarity with biased language was not assessed. We also did not assess long-term skill retention or application in clinical settings. Finally, the workshop was conducted at a single institution for preclinical medical students, limiting generalizability.

### Future Steps

This workshop can be adapted for asynchronous formats and delivered remotely. It may be adapted for use by health care educators, clinicians, and trainees across a variety of health professions. Future research should examine long-term skill retention and the translation of documentation practices into clinical environments, or should incorporate randomized controlled designs or delayed-intervention groups to better isolate intervention effects. Future research may need to shift toward adapting the workshop to include examples of AI-generated notes. The increased use of AI-assisted documentation highlights the relevance of this content; electronic scribes can now draft notes from the audio of clinical encounters, but these systems learn from human-authored records and may reproduce their biased language. On the other hand, studies have shown that these models may increasingly detect and replace stigmatizing language.^20^ In either case, AI-generated documentation requires clinician editing, making the skill of recognizing biased language continue to be critically important.

### Conclusion

As patient access to EHRs continues to expand, attention to language in clinical documentation remains increasingly relevant in the physician-patient relationship. As more of these records are drafted by AI models, the language a physician chooses to write, or chooses to accept, remains a vital human act. By implementing educational interventions early in their training aimed at recognizing biased language, physicians will be equipped with the skills to maintain trust between them and their patients.

## Appendices


Identifying Biased Language in Patient Care.pptxPatient Cases.docxFacilitator Guide.docxPreworkshop Survey.docxPostworkshop Survey.docx
*All appendices are peer reviewed as integral parts of the Original Publication.*
