# Supplementary material for: When Words Shape Care: Educating First-Year Medical Students on Identifying and Replacing Bias in Electronic Health Documentation
Source: MedEdPORTAL. 2026 Oct 29;22:11646. doi: 10.15766/mep_2374-8265.11646 (PMC13717312; doi:10.15766/mep_2374-8265.11646)
Supplement: Supplementary file 1 [file mep_2374-8265.11646-s001.zip › B. Patient Cases.docx]

This document compiles all of the patient case examples used across the pre-survey, workshop, and post-survey in one location. The examples of biased language at the time of document creation are highlighted. As the workshop progresses through future iterations, facilitators can use this document to identify any words that may need to be updated as preferred language evolves, before updating the slide deck and/or surveys accordingly.

Page three includes the three case examples used during the workshop session. Examples of biased language are not highlighted, and this page can be printed out and distributed to participants who may have difficulty reading the slide.

**Identifying & Changing Biased Language in Patient Care - All Patient Cases**

**Case 1: Pre-survey**

28 YOF presents with a 2-week history of fever and persistent cough. Pt states the cough started 2 weeks ago and has persisted since. The fever started 2 days ago and she claims her temperature has consistently been 102F. Temperature taken in the office shows 98.5F. Pt denies nausea and vomiting, but states she had diarrhea the last two days. She is schizophrenic and has a history of hypertension. Her mother committed suicide four years ago and her maternal grandmother is also schizophrenic.

**Case 2: Pre-survey**

36 YOF presents with left forearm pain that started 12 hours ago. She rates her pain 10/10 and claims “this is the worst pain I have ever experienced.” Pt is emotional about the pain impacting her life as she can’t take off from work. Pt is wheelchair bound following a skiing accident at 34 and had an appendectomy at 18. Yesterday afternoon she played tennis for the first time in a year and got herself injured.

**Case 3: Workshop**

16 year-old female (16 YOF) presents with sudden onset severe pain which she rates 10/10. She states the pain is mostly around her chest and upper extremities. Pt has similar episodes every year and is narcotic dependent. Pt is a sickler and often presents at the ER drug seeking. Pt denies any nausea, vomiting, and headache. She claims she took OTC meds w/o relief but has no idea which medication it was.

**Case 4: Post-survey**

This is a presentation by a resident to an attending:

Patient is a 44YOM who came in last night with acute onset of chest pain and difficulty breathing. He claims the pain is 10/10 and denies any radiation. He denies any constipation, nausea, and vomiting. He has no history of heart disease but is an alcoholic. Imaging and EKG are all negative but on rechecking his vitals his pulse ox showed 75%, so he was started on a non-rebreather which he refuses to use. He was started on pain medication, but claims his pain is still 10/10. He also insists he has difficulty breathing when sitting up but is non-compliant with our recommendations to use the non-rebreather.

**Case 5: Post-survey**

24 YOF is brought to the ER by her friends due to aggressive behavior, confusion, and hallucinations. Her friends state that she has been acting “weirdly” for the last three hours. Pt has been talking to imaginary people and was yelling utter nonsense and called the nurses “assholes.” She is a known substance abuser, and her UTOX was positive for PCP.

**Identifying & Changing Biased Language in Patient Care - Workshop Patient Cases**

**Case 1: Pre-survey**

28 YOF presents with a 2-week history of fever and persistent cough. Pt states the cough started 2 weeks ago and has persisted since. The fever started 2 days ago and she claims her temperature has consistently been 102F. Temperature taken in the office shows 98.5F. Pt denies nausea and vomiting, but states she had diarrhea the last two days. She is schizophrenic and has a history of hypertension. Her mother committed suicide four years ago and her maternal grandmother is also schizophrenic.

**Case 2: Pre-survey**

36 YOF presents with left forearm pain that started 12 hours ago. She rates her pain 10/10 and claims “this is the worst pain I have ever experienced.” Pt is emotional about the pain impacting her life as she can’t take off from work. Pt is wheelchair bound following a skiing accident at 34 and had an appendectomy at 18. Yesterday afternoon she played tennis for the first time in a year and got herself injured.

**Case 3: Workshop**

16 year-old female (16 YOF) presents with sudden onset severe pain which she rates 10/10. She states the pain is mostly around her chest and upper extremities. Pt has similar episodes every year and is narcotic dependent. Pt is a sickler and often presents at the ER drug seeking. Pt denies any nausea, vomiting, and headache. She claims she took OTC meds w/o relief but has no idea which medication it was.
