# Supplementary material for: When Words Shape Care: Educating First-Year Medical Students on Identifying and Replacing Bias in Electronic Health Documentation
Source: MedEdPORTAL. 2026 Oct 29;22:11646. doi: 10.15766/mep_2374-8265.11646 (PMC13717312; doi:10.15766/mep_2374-8265.11646)
Supplement: Supplementary file 1 [file mep_2374-8265.11646-s001.zip › C. Facilitator Guide.docx]

**Anti-Bias Workshop with Case Examples as a Tool for Reducing Biased Language in Patient Care**

This document provides detailed instructions for facilitating the Anti-Biased Language Workshop for first-year medical students. It is designed to provide instructors with a background on the topic and the information needed to implement the workshop independently. We recommend that all facilitators read this document several weeks before conducting the workshop to familiarize themselves with the flow of the session and prepare for any tasks, including finding a suitable room and sending out the pre-survey. It is also recommended that facilitators have this document with them during the workshop to easily refer to the order of session activities.

**Background and Context of the Workshop**

Healthcare disparities persist across the United States and continue to contribute to adverse patient outcomes and negative patient experiences. One mechanism by which these disparities are perpetuated is the use of biased and stigmatizing language in medical documentation in the Electronic Health Record (EHR), which may characterize patients based on race, medical condition, disability, or other social and clinical attributes. Research has increasingly demonstrated that biased language in medical records influences both provider perceptions and clinical decision-making and can have a significant impact on patient care. Although medical school curricula increasingly emphasize diversity, equity, and bias recognition, learners may continue to adopt stigmatizing documentation practices during professional identity formation. As a result, educators have called for earlier and formal instruction focused on humanism, ethics, and patient-centered communication.

This structured charting workshop for first-year medical students is focused on identifying biased language in medical records and recognizing patient-centered alternatives. It emphasizes the impact of biased documentation on patient care and provider perceptions and utilizes case-based discussion to facilitate active learning. Our goal is to equip students with the skills necessary to recognize and avoid biased language before entering clinical rotations. Although designed for preclinical medical students, this workshop may be adapted for use by healthcare educators, clinicians, and trainees across a variety of health professions.

**Educational Objectives:**

By the end of this activity, learners will be able to:

1. Demonstrate increased awareness of biased language in medical documentation and its potential impact on patient care and provider perceptions.
2. Identify stigmatizing or biased language commonly used in medical documentation.
3. Recognize patient-centered, neutral, and acceptable alternatives to biased language in clinical notes.

**Practical Information**

Approximately one week prior to hosting the workshop, facilitators should send out the pre-survey **(Appendix D)** to all potential attendees.

The outlined workshop agenda below is to be used as a facilitator’s guide in tandem with **Appendix A**, the slide presentation. Detailed presentation scripts can be found in the comments section of each slide in the presentation. Instructors may use this script word-for-word or add additional information if they feel comfortable. It is recommended that the room chosen for the workshop session have a projector screen or large television.

Instructors should display the slides on a large screen before starting the workshop presentation. We recommend that 2-3 presenters facilitate the workshop.

During the workshop, participants will be asked to get into small groups of 3-5 people. Participants will also speak in a large group format; therefore, it is recommended that workshop attendance be capped at approximately 80 people.

A full list of sample patient chart cases used throughout the activity can be found in **Appendix B**. If the session has a large number of participants, the last page of that document can be printed and distributed to each group for ease of reading.

| Time (mins) | Activity |
| --- | --- |
| 5 | **Introduction (Appendix A)**  Introduce the workshop’s educational objectives (Slides 1-2).  Define biased language (Slide 3) and describe the broad types (Slide 4).  Discuss the effects of biased language (Slides 5-7).  Then, for 2-3 minutes, have the audience discuss with surrounding colleagues, “What examples have you witnessed of clinicians using biased language (in-person or in the EMR)?”  Ask for volunteers to share their reflections with the group. *If there are no volunteers, facilitators can take this time to share their own experiences.* |
| 15 | **Do’s & Don’ts (Appendix A)**  Describe common forms of biased language and preferred alternatives (Slides 8-11). State the language to avoid, followed by the preferred alternative so that students can make the association. Then, move on to the next example of language to avoid.  Pause after each slide for any questions the audience may have. |
| 10 | **Small Group Activity and Large Group Discussion #1 (Appendix A and B)**  The audience will divide into small groups of 3-5 people.  Facilitators will display Case #1 on the screen and read it out loud, then instruct small groups to discuss for 5 minutes and identify: (Slides 12-13)   - What language used in the case could be considered biased? - How would you change the language to reduce bias?   Ask for volunteers to share their responses to the above questions with the whole group.  Then, point out examples of biased language in the case as highlighted and briefly discuss why each is biased (Slide 14). If students previously pointed out language that would not be considered biased, take a moment to explain why that language would be considered acceptable.  Facilitators will then show the same case rewritten with unbiased language (Slide 15).  Pause to answer any audience questions.  The audience will then return to their previous small group. |
| 10 | **Small Group Activity and Large Group Discussion #2 (Appendix A and B)**  Facilitators will display Case #2 on the screen and read it out loud, then instruct small groups to discuss for 5 minutes and identify: (Slides 16)   - What language used in the case could be considered biased? - How would you change the language to reduce bias?   Ask for volunteers to share their responses to the above questions with the whole group.  Then, point out examples of biased language in the case as highlighted and briefly discuss why each is biased (Slide 17). If students previously pointed out language that would not be considered biased, take a moment to explain why that language would be considered acceptable.  Facilitators will then show the same case rewritten with unbiased language (Slide 18).  Pause to answer any audience questions.  The audience will then return to their previous small group. |
| 10 | **Small Group Activity and Large Group Discussion #3 (Appendix A and B)**  Facilitators will display Case #3 on the screen and read it out loud, then instruct small groups to discuss for 5 minutes and identify: (Slides 19)   - What language used in the case could be considered biased? - How would you change the language to reduce bias?   Ask for volunteers to share their responses to the above questions with the whole group.  Then, point out examples of biased language in the case as highlighted and briefly discuss why each is biased (Slide 20). If students previously pointed out language that would not be considered biased, take a moment to explain why that language would be considered acceptable.  Facilitators will then show the same case rewritten with unbiased language (Slide 21).  Pause to answer any audience questions.  As this was the last section of the small group activity, students can remain as part of the large group for the next section. |
| 10 | **Conclusion (Appendix A and E)**  Summarize key takeaways from the workshop. Encourage students to learn from other healthcare professionals and to be aware of positive and negative language they may encounter in the EHR (Slide 22).  The post-survey (**Appendix E**) can be distributed for completion before leaving the session (Slide 23). |
