# Supplementary material for: When Words Shape Care: Educating First-Year Medical Students on Identifying and Replacing Bias in Electronic Health Documentation
Source: MedEdPORTAL. 2026 Oct 29;22:11646. doi: 10.15766/mep_2374-8265.11646 (PMC13717312; doi:10.15766/mep_2374-8265.11646)
Supplement: Supplementary file 1 [file mep_2374-8265.11646-s001.zip › D. Preworkshop Survey.docx]

This document includes all of the pre-survey questions, including a unique participant identifier, demographic information, two patient case examples, and three general knowledge questions. The two case examples will be the first two cases discussed during the workshop, providing participants an opportunity to reflect on what they have learned.

We recommend that facilitators convert these questions to an online survey tool, such as Qualtrics, for ease of survey distribution and data analysis.

**Identifying & Changing Biased Language in Patient Care - Workshop Pre-Survey**

**Creating your unique ID**

Please create your unique ID using the following: First three letters of your mother’s maiden name and first three letters of mother’s birth month - **ALL CAPITAL LETTERS.**

This will be the same ID you input for the post-surveys.

Please select your gender:

☐ Man

☐ Woman

☐ Transgender man

☐ Transgender woman

☐ Non-binary/non-conforming

☐ Prefer not to respond

☐ Other:

Please select your race/ethnicity:

(Select all that apply)

☐ American Indian or Alaska Native

☐ Asian

☐ Black or African American

☐ Hispanic or Latino

☐ Middle Eastern or North African

☐ Native Hawaiian or Pacific Islander

☐ White

☐ Other:

**Case 1**

Case 1:

28 YOF presents with a 2-week history of fever and persistent cough. Pt states the cough started 2 weeks ago and has persisted since. The fever started 2 days ago and she claims her temperature has consistently been 102F. Temperature taken in the office shows 98.5F. Pt denies nausea and vomiting, but states she had diarrhea the last two days. She is schizophrenic and has a history of hypertension. Her mother committed suicide four years ago and her maternal grandmother is also schizophrenic.

Select whether you believe the sentences from the case above are appropriate or inappropriate to be included in a patient's chart.

|  | Appropriate | Not Appropriate | Unsure |
| --- | --- | --- | --- |
| 28 YOF presents with a 2-week history of fever and persistent cough. |  |  |  |
| Pt states the cough started 2 weeks ago and has persisted since. |  |  |  |
| The fever started 2 days ago and she claims her temperature has consistently been 102F. |  |  |  |
| Temperature taken in the office shows 98.5F. |  |  |  |
| Pt denies nausea and vomiting, but states she had diarrhea the last two days. |  |  |  |
| She is schizophrenic and has a history of hypertension. |  |  |  |
| Her mother committed suicide four years ago and her maternal grandmother is also schizophrenic. |  |  |  |

**Case 2**

Case 2:

36 YOF presents with left forearm pain that started 12 hours ago. She rates her pain 10/10 and claims “this is the worst pain I have ever experienced.” Pt is emotional about the pain impacting her life as she can’t take off from work. Pt is wheelchair bound following a skiing accident at 34 and had an appendectomy at 18. Yesterday afternoon she played tennis for the first time in a year and got herself injured.

Select whether you believe the sentences from the case above are appropriate or inappropriate to be included in a patient's chart.

|  | Appropriate | Not Appropriate | Unsure |
| --- | --- | --- | --- |
| 36 YOF presents with left forearm pain that started 12 hours ago. |  |  |  |
| She rates her pain 10/10 and claims “this is the worst pain I have ever experienced.” |  |  |  |
| Pt is emotional about the pain impacting her life as she can’t take off from work. |  |  |  |
| Pt is wheelchair bound following a skiing accident at 34 and had an appendectomy at 18. |  |  |  |
| Yesterday afternoon she played tennis for the first time in a year and got herself injured. |  |  |  |

**General Knowledge Questions**

Please mark the following statements as true, false, or unsure.

|  | True | False | Unsure |
| --- | --- | --- | --- |
| Biased language in notes can harm the physician-patient relationship. |  |  |  |
| Unbiased language makes notes more difficult to understand. |  |  |  |
| Stigmatizing language can influence the future of a patient's care. |  |  |  |

Which of the following people are able to access and read a patient's note? Select all that apply.

☐ The doctor on the case

☐ Residents on the case

☐ The patient

☐ Future physicians involved in the patient’s care

What kind/type of bias do you think is most commonly seen in a patient’s note?
