# Supplementary material for: When Words Shape Care: Educating First-Year Medical Students on Identifying and Replacing Bias in Electronic Health Documentation
Source: MedEdPORTAL. 2026 Oct 29;22:11646. doi: 10.15766/mep_2374-8265.11646 (PMC13717312; doi:10.15766/mep_2374-8265.11646)
Supplement: Supplementary file 1 [file mep_2374-8265.11646-s001.zip › E. Postworkshop Survey.docx]

This document includes all of the post-survey questions, including a unique participant identifier, two patient case examples, three general knowledge questions, and three workshop feedback questions. The two case examples are unique from the cases discussed during the workshop. The general knowledge questions are the same as in the pre-survey to allow for assessment of knowledge. The workshop feedback questions allow participants to reflect on what they learned and suggest any improvements.

We recommend that facilitators convert these questions to an online survey tool, such as Qualtrics, for ease of survey distribution and data analysis.

**Identifying & Changing Biased Language in Patient Care - Workshop Post-Survey**

**Creating your unique ID**

Please create your unique ID using the following: First three letters of your mother’s maiden name and first three letters of mother’s birth month - **ALL CAPITAL LETTERS!**

This will be the same ID you entered for the pre-survey.

**Case 1**

Case 1:

This is a presentation by a resident to an attending:

Patient is a 44YOM who came in last night with acute onset of chest pain and difficulty breathing. He claims the pain is 10/10 and denies any radiation. He denies any constipation, nausea, and vomiting. He has no history of heart disease but is an alcoholic. Imaging and EKG are all negative but on rechecking his vitals his pulse ox showed 75%, so he was started on a non-rebreather which he refuses to use. He was started on pain medication, but claims his pain is still 10/10. He also insists he has difficulty breathing when sitting up but is non-compliant with our recommendations to use the non-rebreather.

Select whether you believe the sentences from the case above are appropriate or inappropriate to be included in a patient's chart.

|  | Appropriate | Not Appropriate | Unsure |
| --- | --- | --- | --- |
| Patient is a 44YOM who came in last night with acute onset chest pain and difficulty breathing. |  |  |  |
| He claims the pain is 10/10 and denies any radiation. |  |  |  |
| He denies any constipation, nausea, and vomiting. |  |  |  |
| He has no history of heart disease but is an alcoholic. |  |  |  |
| Imaging and EKG are all negative but on rechecking his vitals his pulse ox showed 75%, so he was started on a non-rebreather which he refuses to use. |  |  |  |
| He was started on pain medication, but claims his pain is still 10/10. |  |  |  |
| He also insists he has difficulty breathing when sitting up but is non-compliant with our recommendations to use the non-rebreather. |  |  |  |

**Case 2**

Case 2:

24 YOF is brought to the ER by her friends due to aggressive behavior, confusion, and hallucinations. Her friends state that she has been acting “weirdly” for the last three hours. Pt has been talking to imaginary people and was yelling utter nonsense and called the nurses “assholes.” She is a known substance abuser, and her UTOX was positive for PCP.

Select whether you believe the sentences from the case above are appropriate or inappropriate to be included in a patient's chart.

|  | Appropriate | Not Appropriate | Unsure |
| --- | --- | --- | --- |
| 24 YOF is brought to the ER by her friends due to aggressive behavior, confusion, and hallucinations. |  |  |  |
| Her friends state that she has been acting “weirdly” for the last three hours. |  |  |  |
| Pt has been talking to imaginary people and was yelling utter nonsense and called the nurses “assholes.” |  |  |  |
| She is a known substance abuser, and her UTOX was positive for PCP. |  |  |  |

**General Knowledge Questions**

Please mark the following statements as true, false, or unsure.

|  | True | False | Unsure |
| --- | --- | --- | --- |
| Biased language in notes can harm the physician-patient relationship. |  |  |  |
| Unbiased language makes notes more difficult to understand. |  |  |  |
| Stigmatizing language can influence the future of a patient's care. |  |  |  |

Which of the following people are able to access and read a patient's note? Select all that apply.

☐ The doctor on the case

☐ Residents on the case

☐ The patient

☐ Future physicians involved in the patient’s care

**Rate your experience**

Rate the workshop and what you have learned.

|  | Strongly agree | Somewhat agree | Neutral | Somewhat disagree | Strongly disagree |
| --- | --- | --- | --- | --- | --- |
| This workshop was helpful. |  |  |  |  |  |
| I have a better understanding of what is biased and unbiased language. |  |  |  |  |  |
| I understand why biased language can impact a patient's care even years down the line. |  |  |  |  |  |

Please list one thing you learned from this workshop.

Please provide any thoughts or comments on how this workshop could be improved for future sessions.
